# Post-translational Modifications near the Quinone Binding Site of Mammalian Complex I*[Fn FN2]

**DOI:** 10.1074/jbc.M113.488106

**Published:** 2013-07-08

**Authors:** Joe Carroll, Shujing Ding, Ian M. Fearnley, John E. Walker

**Affiliations:** From the Mitochondrial Biology Unit, Medical Research Council, Hills Road, Cambridge CB2 0XY, United Kingdom

**Keywords:** Bioenergetics/Electron Transfer Complex, Electron Transport System (ETS), Mitochondria, Protein Chemical Modification, Protein Methylation, Arginine Modification, Complex I, Protein Hydroxylation

## Abstract

Complex I (NADH:ubiquinone oxidoreductase) in mammalian mitochondria is an L-shaped assembly of 44 protein subunits with one arm buried in the inner membrane of the mitochondrion and the orthogonal arm protruding about 100 Å into the matrix. The protruding arm contains the binding sites for NADH, the primary acceptor of electrons flavin mononucleotide (FMN), and a chain of seven iron-sulfur clusters that carries the electrons one at a time from FMN to a coenzyme Q molecule bound in the vicinity of the junction between the two arms. In the structure of the closely related bacterial enzyme from *Thermus thermophilus*, the quinone is thought to bind in a tunnel that spans the interface between the two arms, with the quinone head group close to the terminal iron-sulfur cluster, N2. The tail of the bound quinone is thought to extend from the tunnel into the lipid bilayer. In the mammalian enzyme, it is likely that this tunnel involves three of the subunits of the complex, ND1, PSST, and the 49-kDa subunit. An arginine residue in the 49-kDa subunit is symmetrically dimethylated on the ω-N^G^ and ω-N^G′^ nitrogen atoms of the guanidino group and is likely to be close to cluster N2 and to influence its properties. Another arginine residue in the PSST subunit is hydroxylated and probably lies near to the quinone. Both modifications are conserved in mammalian enzymes, and the former is additionally conserved in *Pichia pastoris* and *Paracoccus denitrificans*, suggesting that they are functionally significant.

## Teaser

**Background:** Complex I in mammalian mitochondria is an assembly of 44 proteins.

**Results:** An arginine residue in the 49-kDa subunit is symmetrically dimethylated, and another arginine in the PSST subunit is hydroxylated.

**Conclusion:** As the modifications are conserved and lie close to the electron transfer pathway, they are probably functionally significant.

**Significance:** The chemical composition of mammalian complex I has been completely defined.

## Introduction

Mammalian respiratory complex I (NADH:ubiquinone oxidoreductase) is one of the most complex enzymes yet described. It is an L-shaped assembly of 44 proteins with a combined mass of about 1 MDa, with one arm buried in the inner membrane of the mitochondrion, and the orthogonal arm protruding about 100 Å into the matrix of the organelle (1–4). This membrane extrinsic arm contains the binding sites for NADH and the primary acceptor of electrons FMN, and it provides a scaffold for a chain of seven iron-sulfur clusters that carries the electrons one at a time from FMN through the extrinsic arm to a coenzyme Q molecule bound in the vicinity of the junction between the peripheral and membrane arms (5). The process of electron transfer reduces the coenzyme Q molecule and provides the energy to translocate protons from the matrix of the mitochondrion, through the membrane domain of the enzyme, into the intermembrane space of the organelle, thereby contributing to the proton motive force. For each two electrons transferred from NADH to coenzyme Q, four protons are ejected from the mitochondrial matrix (6, 7).

The protein subunit composition of the bovine enzyme has been characterized most extensively (2, 8), and it serves as a model for the human enzyme (9). Seven of the 44 subunits of both bovine and human enzymes are translated in the matrix from their mitochondrial genomes, and the remainder are the products of nuclear genes that are imported into the organelle. The extrinsic arm contains 23 of these nuclear encoded subunits. Seven of them (75 kDa, 51 kDa, 49 kDa, 30 kDa, 24 kDa, PSST, and TYKY) (see “Experimental Procedures” for nomenclature of subunits) form the catalytic core of the enzyme, which binds NADH, FMN, and the iron-sulfur clusters, and the remainder are supernumerary subunits that are not involved directly in catalysis (10, 11). The seven mitochondrially encoded subunits form the core of the membrane arm and are folded into four domains that resemble antiporter proteins (5). These antiporter domains probably provide the pathways for the translocation of protons through the inner membrane. During the detailed characterization of the subunits of the bovine enzyme, it has been demonstrated that the mitochondrial gene products assembled in complex I retain *N*α-formyl groups on their translational initiator methionine residues, but there are no stable post-translational modifications (12, 13), and with the exception of the phosphorylation of the murine ND5 subunit of complex I (14), no transient modifications have been noted so far. In contrast, the nuclear encoded subunits contain many post-translational modifications, both transient and stable (15). The transient modifications include partial phosphorylation of the 42-kDa, ESSS, MWFE, B14.5a, B14.5b, and B16.6 subunits (16–18) and N-ϵ-acetylation of specific lysine residues in the 75-kDa, 51-kDa, 42-kDa, 39-kDa, 13-kDa, B17.2, B13, B12, and B8 subunits (19). The stable modifications include the removal of mitochondrial import sequences from 18 subunits and the introduction of iron-sulfur clusters into five of them (the 75-kDa, 51-kDa, 24-kDa, PSST, and TYKY subunits). Translational initiator methionines are removed from 16 of the subunits lacking processed import sequences, with the *N*α-acetylation of residue 2 of all but five of these proteins and the *N*α-myristoylation of a 12th, subunit B18. In addition, the initiator methionine residues of two subunits (B17.2 and B14.5b) are acetylated (20, 21). The mature SDAP subunit, which serves as an acyl-carrier protein, carries a phosphopantetheine moiety attached to residue 44 (22). Finally, subunit B12, which is partially *N*α-acetylated, has a complex pattern of methylation of unknown significance on histidines 4, 6, and 8 (15).

As described here, we have sought an explanation of two discrepancies between the experimentally determined and calculated molecular masses of the 49-kDa and PSST subunits, both being components of the extrinsic arm of bovine complex I. Their experimentally determined values exceed those calculated from their sequences by about 28 and 16 daltons, respectively (2, 23). The explanation provided below is that both subunits contain unusual post-translational modifications of specific arginine residues that are close to functionally important sites in complex I.

## EXPERIMENTAL PROCEDURES

### 

#### 

##### Nomenclature of Subunits of Bovine Complex I

The nomenclature of subunits of bovine complex I has been explained previously (10). Accordingly, subunits PSST and so forth are named in one-letter amino acid code of the sequences of residues 1–4 at their N termini, and other subunits (*e.g.* 49-kDa subunit) are named from their apparent molecular masses.

##### Preparation of Complexes I

Bovine complex I was isolated from heart mitochondria (8, 24), and the human enzyme was isolated from human embryonic kidney cells (HEK 293-F). The cells were grown in suspension at 37 °C under an atmosphere of 8% CO_2_ in CD293 medium (Life Technologies Ltd., Paisley, UK) containing 1× GlutaMAX (Life Technologies), penicillin (100 units/ml), and streptomycin (0.1 mg/ml). Mitochondria were prepared as described before (25) except that the DNase I treatment was omitted. Complex I was recovered from HEK mitochondria by immunocapture (Abcam, Cambridge, UK). Tryptic digests of the Nqo4 subunit of complex I from *Paracoccus denitrificans* and of intact complex from *Pichia pastoris* were prepared as described before (26, 27).

##### Characterization of Subunits of Complexes I

Samples of bovine, human, and *E. coli* complexes I were reduced with tris(2-carboxyethyl)phosphine (5 mm, 30 min, 37 °C) and alkylated with iodoacetamide (15 mm, 30 min) in SDS gel sample buffer at pH 8.0. The alkylated proteins were fractionated by SDS-PAGE in Tris-glycine buffer in 10–20% gradient gels (Life Technologies) and stained with Coomassie Blue dye. The stained bands were excised and digested in-gel separately with trypsin, chymotrypsin, and Asp-N (Roche Applied Science, Burgess Hill, UK (28)). Subunits were identified by mass spectrometric analyses of the proteolytic digests. Samples containing the bovine 49-kDa subunit were isolated from subcomplexes Iα and Iλ by reverse-phase chromatography (2) and digested with either trypsin or Asp-N protease. Peptide mixtures were analyzed in a MALDI-TOF-TOF mass spectrometer (model 4800; AB Sciex, Warrington, UK) with α-cyano-4-hydroxycinnamic acid as matrix where they were fragmented by collision-induced dissociation (CID)^2^ with air. Alternatively, they were fractionated by reverse-phase chromatography using a Proxeon EASY-nLC (Thermo Fisher, Hemel Hempstead, UK) for nanoscale (75-μm inner diameter × 100-mm C_18_ column; Nanoseparations, Nieuwkoop, The Netherlands) reverse-phase peptide separation using an acetonitrile gradient in 0.1% (v/v) formic acid at 300 nl/min, and the column effluent was introduced on-line into an LTQ Orbitrap XL-ETD (electron transfer dissociation) mass spectrometer (Thermo Fisher). Peptides were fragmented by CID with nitrogen, by high energy collision dissociation (HCD (29)) with nitrogen, or by electron transfer dissociation (ETD) with fluoranthene radical anions and supplemental activation (30). Peptides were identified from Orbitrap and MALDI-TOF-TOF CID fragmentation data by comparison with NCBInr protein sequence databases. MALDI-TOF-TOF data were analyzed with Mascot (Matrix Science Ltd., London, UK) with the following parameters: NCBInr-mammals; precursor ion mass tolerance 70 ppm; fragment ion mass tolerance 0.8 Da; Met oxidation variable, Cys-carbamidomethyl fixed; trypsin 2-missed, Asp-N_ambic 3-missed, chymotrypsin 4-missed cleavages. The significance threshold for peptide identification was *p* < 0.05. Orbitrap peptide fragmentation data were analyzed using Proteome Discoverer 1.3 (Thermo Fisher) with Mascot and Peptide Validator nodes. The following parameters were employed: NCBInr-mammals; precursor ion mass tolerance 5 ppm; fragment ion mass tolerance 0.5 Da; Met oxidation variable, Cys-carbamidomethyl fixed (in-gel digests) or no Cys modification (in-solution digests); trypsin 2-missed, Asp-N_ambic 3-missed, chymotrypsin 4-missed cleavages; decoy database search (false discovery rate values 0.01 and 0.05). Data from the fragment ion spectra of selected post-translationally modified peptides were interpreted manually. The presence and relative abundance of modified *versus* unmodified peptides were estimated with Xcalibur software from peak area calculations of Gaussian smoothed extracted ion chromatograms, applying an *m*/*z* tolerance of 5 ppm.

## RESULTS

### 

#### 

##### Reinvestigation of the Sequences of the 49-kDa and PSST Subunits of Bovine Complex I

**FIGURE 1.**
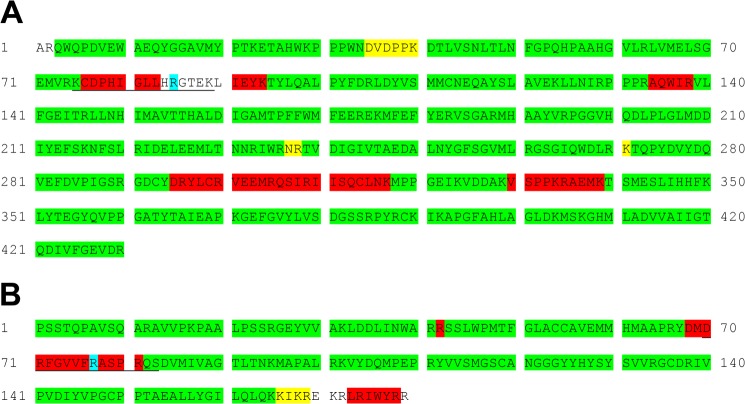
**Reinvestigation of the sequences of the 49-kDa and PSST subunits of bovine complex I.** The sequences of peptides in proteolytic digests of the proteins were analyzed by tandem mass spectrometry. *A*, the 49-kDa subunit; *B*, the PSST subunit. Segments highlighted in *green*, *yellow*, and *red* correspond to regions where the sequence was confirmed with high, medium, or low confidence, respectively. No evidence was found in these experiments for the regions that are not highlighted. In subsequent analyses presented below, a tryptic peptide corresponding to residues 75–89 of the 49-kDa subunit (*underlined*) was found to be post-translationally modified by symmetrical dimethylation of the guanidino group of residue Arg-85, and an Asp-N peptide from the PSST subunit corresponding to residues 70–83 (*underlined*) was shown to bear a hydroxyl group on residue Arg-77. The modified residues are highlighted in *light blue*. In the 49-kDa subunit, both arginine and glutamine have been found at residue 129.

**FIGURE 2.**
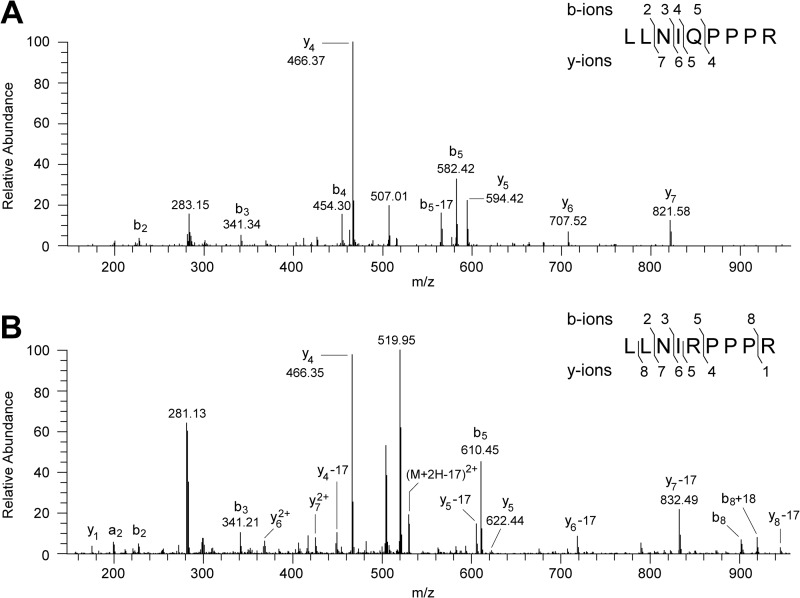
**Evidence for the presence of two isoforms of the 49-kDa subunit in preparations of bovine complex I.** The isoforms differ by the presence of arginine or glutamine at position 129 of the mature protein. *A* and *B*, fragment ion spectra produced by CID of doubly charged ions with *m*/*z* 524.32 and 538.34 arising from the tryptic peptides corresponding to residues 125–133 of the 49-kDa subunit. The fragment ions y_4_-y_5_ and b_4_-b_5_ define residue 129 as Gln in *A* and as Arg in *B*, in the tryptic peptide sequence LLNI(Q/R)PPPR. In *B*, the y-ions appear as the y-ion series and as a y-17 series arising from the loss of ammonia. In the *insets*, the identified b- and y- fragment ions are mapped onto the amino acid sequence.

The mass spectrometric analysis of enzymic digests of both the 49-kDa and the PSST subunits with trypsin, chymotrypsin, and protease Asp-N validated almost the entire amino acid sequences of both proteins with high or medium confidence (Fig. 1). However, residues 76–94, 295–317, and 330–339 in the 49-kDa subunit and 68–81 and 170–179 in the PSST subunit were either not covered at all (residues 84–90 in the 49-kDa subunit and 170–172 and 179 in the PSST subunit), or they were covered sparsely. Therefore, these regions became the focus of attention in the search for an explanation of the discrepancies between experimentally measured and calculated intact protein masses. An additional complexity in the case of the bovine 49-kDa subunit is that there are two isoforms differing by the single amino acid substitution R129Q, presumably arising from different alleles in the bovine population (31). Tryptic peptides corresponding to both isoforms were detected (Fig. 2), but the predominant component, as estimated from the areas of the ion peaks, was the isoform with Arg-129. The calculated masses of the isoforms of the 49-kDa subunit with Arg-129 and Gln-129 are 28 and 56 Da, respectively, less than the measured intact molecular mass (2, 23).

##### The Post-translational Modification of the 49-kDa Subunit of Complex I

**FIGURE 3.**
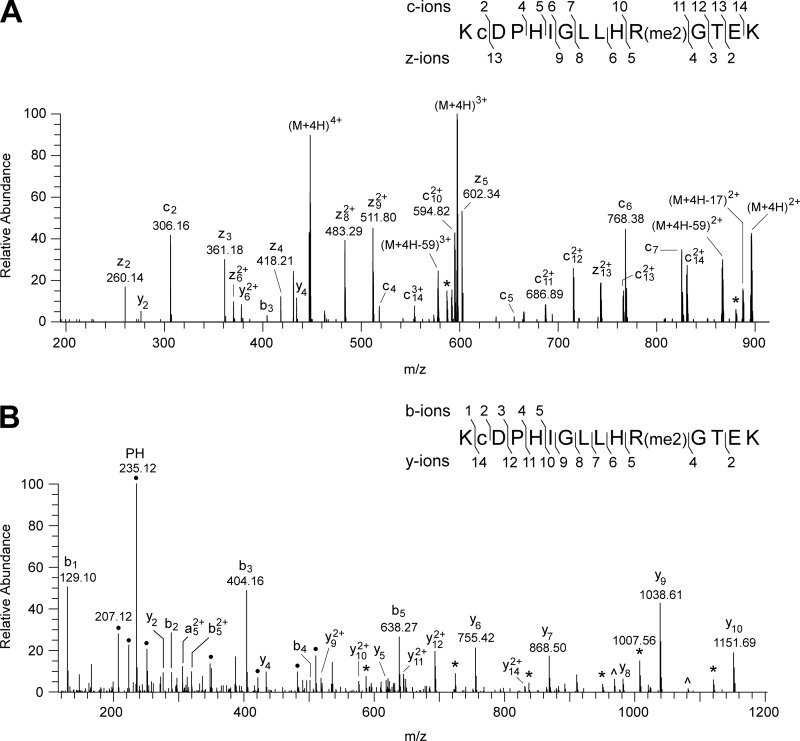
**Characterization of the symmetrical dimethylation of residue Arg-85 of the 49-kDa subunit of bovine complex I.** *A* and *B*, spectra of fragments produced, respectively, by ETD of a quadruply charged ion (447.99 *m*/*z*) and by HCD of a triply charged ion (596.99 *m*/*z*), both derived from the tryptic peptide corresponding to residues 75–89 of the 49-kDa subunit. In *A*, a singly charged ion (447.12 *m*/*z*) is a contaminant of the precursor (M+4H)^4+^ ion. In *B*, ● denotes ions arising by internal fragmentation. The ions z_4_-z_5_, c_10_-c_11_, and y_4_-y_5_ demonstrate dimethylation of residue Arg-85 of the 49-kDa subunit. The losses of the neutral fragments with masses of 31 (monomethylamine) from y_5_-y_10_ and 70 (dimethylcarbodiimide) from y_9_ and y_10_, labeled * and ∧ respectively, define the modification as the symmetrical dimethylation of the guanidino group of residue Arg-85. In the *insets*, the fragment ions are mapped onto the amino acid sequence; *c* is carbamidomethylcysteine.

**FIGURE 4.**
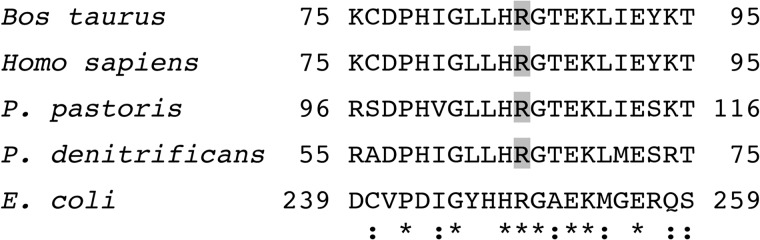
**Comparison of the sequence of the region surrounding the dimethylated arginine residue in the bovine 49-kDa subunit of complex I with orthologous sequences.** Residues 75–95 of the bovine protein were aligned with ClustalW with related sequences from the 49-kDa subunits of the human, *P. pastoris*, and *P. denitrificans* enzymes and subunit NuoCD from the *E. coli* enzyme. The symbols * and : denote identical and conserved residues, respectively. The *shaded arginine residues* are modified by symmetrical dimethylation.

**FIGURE 5.**
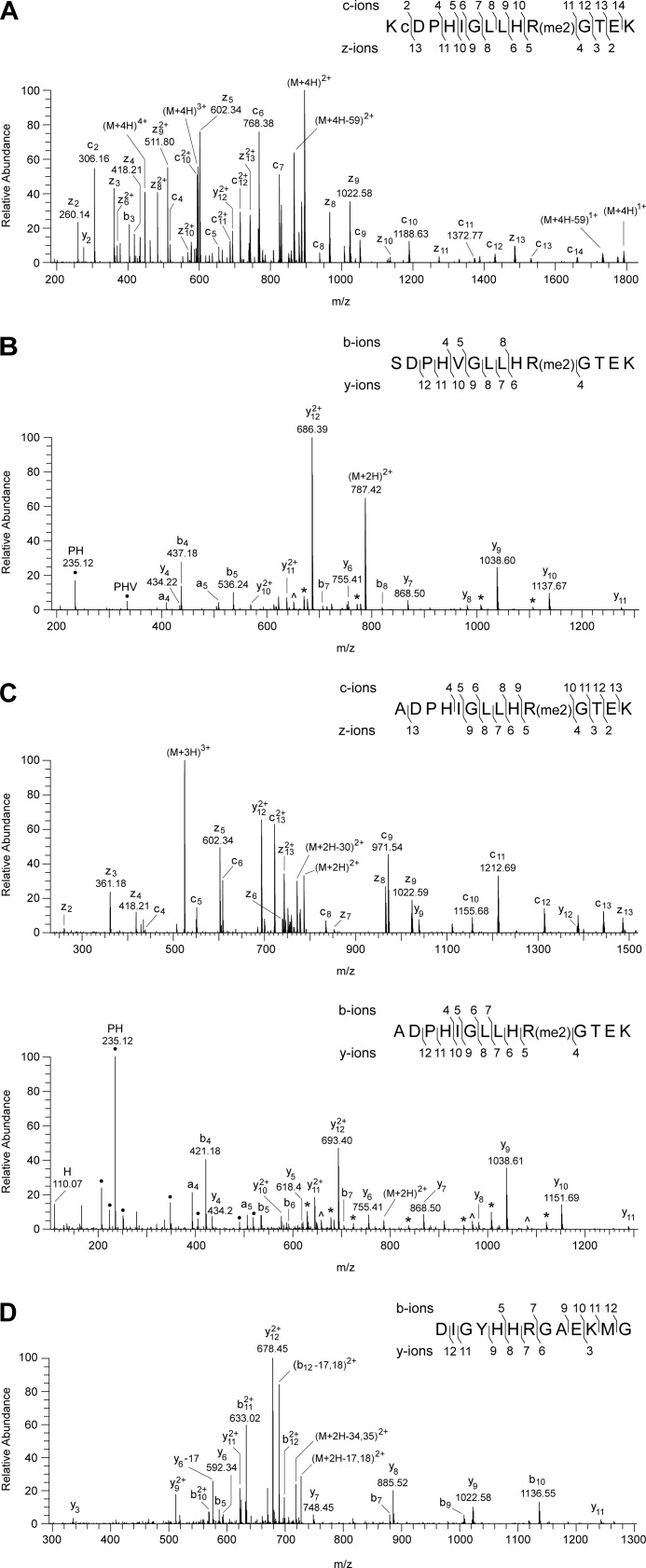
**Characterization of the symmetrical dimethylation of arginine residues of the 49-kDa subunits of complexes I from humans, *P. pastoris*, and *P. denitrificans*.** *A–C* show mass spectrometric analyses of the human, *P. pastoris*, and *P. denitrificans* proteins, and *D* shows that the modification is not found in the equivalent position in the *E. coli* protein. In the *insets*, the fragment ions observed in the spectra are mapped onto the amino acid sequence of the peptides. In the *inset* in *A*, *c* is carbamidomethylcysteine. In *B* and *C*, neutral losses of fragments of 31 (monomethylamine) and 70 (dimethylcarbodiimide) are labeled * and ∧, respectively. They confirm the modification as symmetrical dimethylation of Arg-106 and Arg-65, respectively; ● denotes ions arising by internal fragmentation. *A*, spectrum of fragments produced by ETD from a quadruply charged ion (*m*/*z* 447.99) from the tryptic peptide corresponding to residues 75–89 of the human subunit. The z_4_-z_5_ and c_10_-c_11_ ions demonstrate dimethylation of residue Arg-85. A background 447.12 *m*/*z* singly charged ion is observed as a contaminant of the precursor (M+4H)^4+^ ion in the ETD spectrum. *B*, spectrum of fragments produced by HCD from a doubly charged ion (*m*/*z* 787.42) from the tryptic peptide corresponding to residues 97–110 of the *P. pastoris* subunit. The ions y_4_ and y_6_ define the sequence His-105-dimethyl-Arg-106. *C*, *upper panel*, spectrum of fragments produced by ETD of a triply charged ion (*m*/*z* 524.63); *lower panel*, spectrum of fragments produced by HCD of a doubly charged ion (*m*/*z* 786.44) of the tryptic peptide corresponding to residues 56–69 of the *P. denitrificans* subunit. The z_4_-z_5_, c_9_-c_10_, and y_4_-y_5_ ions demonstrate dimethylation of residue Arg-65. *D*, spectrum of fragments produced by CID from a doubly charged ion (*m*/*z* 735.85) from a peptide corresponding to residues 243–255 of the *E. coli* NuoCD subunit. The series of fragment ions identifies the peptide and excludes modification of residue Arg-249.

In the tryptic digest of the 49-kDa subunit, an unexplained peptide with *m*/*z* value of 1788.95 was discovered by MALDI-TOF analysis. Its mass corresponds to the calculated mass of the tryptic peptide from residues 75–89 plus 28 Da. Fragmentation of this peptide (Fig. 3) confirmed that its sequence, KCDPHIGLLHRGTEK, corresponds to residues 75–89 of the 49-kDa protein with the additional mass of 28 Da associated with residue Arg-85, suggesting that its guanidino group is dimethylated. The fragment ion spectra of the peptide (Fig. 3) contained several y-ions resulting from the neutral loss of 31 and 70 mass units, corresponding to the loss of methylamine and dimethylcarbodiimide, respectively, which are diagnostic of two methyl groups attached symmetrically to the ω-N^G^ and ω-N^G′^ nitrogen atoms of the guanidino group of an arginine residue (32, 33). Thus, it was concluded that residue Arg-85 of the 49-kDa subunit of bovine complex I is symmetrically dimethylated. There was no evidence in the mass spectrum of y-ions arising from the loss of 45 Da corresponding to dimethylamine, which would be diagnostic of an arginine residue asymmetrically dimethylated on one of the Nω nitrogen atoms (32, 33). Also, none of the spectra contained evidence for the unmethylated or monomethylated forms of residue Arg-85, and so the residue appears to be completely symmetrically dimethylated. The equivalent arginine residues in the human, *P. pastoris*, *P. denitrificans*, and *E. coli* enzymes are conserved (Fig. 4). The symmetrical dimethylation of the arginine residue is also conserved in the human, *P. pastoris*, and *P. denitrificans* complexes I (Fig. 5), but the equivalent residue in the NuoCD subunit of the *E. coli* enzyme is not modified (Fig. 5).

##### Post-translational Modification of the PSST Subunit of Complex I

**FIGURE 6.**
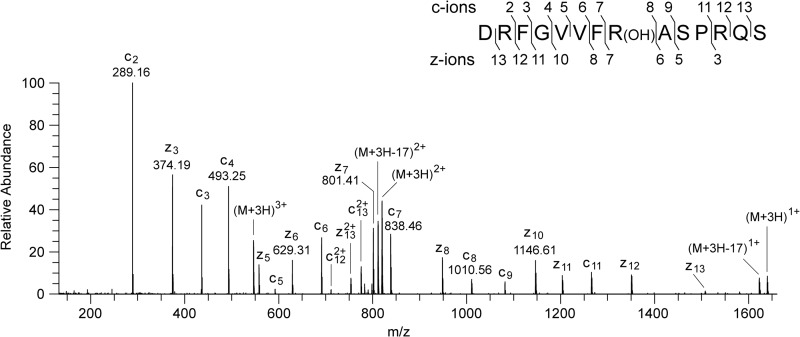
**Characterization of the hydroxylation of residue Arg-77 of the PSST subunit of bovine complex I.** A spectrum of fragments produced by ETD of a triply charged ion with *m*/*z* 546.62 generated from an Asp-N peptide corresponding to residues 70–83 of the PSST subunit is shown. The ions z_6_-z_7_ and c_7_-c_8_ show that the +16-Da modification is associated with residue Arg-77 of the PSST subunit. In the *inset*, the fragment ions are mapped onto the amino acid sequence.

**FIGURE 7.**
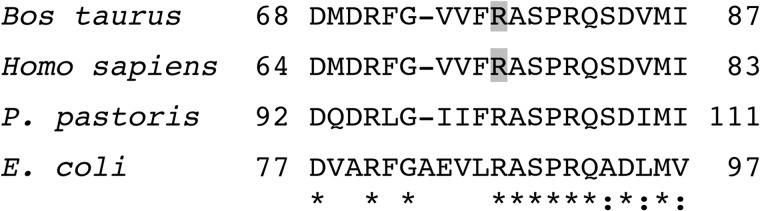
**Comparison of the sequence of the region surrounding the hydroxylated arginine residue in the PSST subunit of bovine complex I with orthologous sequences.** Residues 68–87 of the bovine protein were aligned with ClustalW with related sequences from the PSST subunits of the human, *P. pastoris*, and *E. coli* enzymes. The symbols * and : correspond to identical and conserved residues respectively. The hydroxylated arginine residue is *shaded*.

**FIGURE 8.**
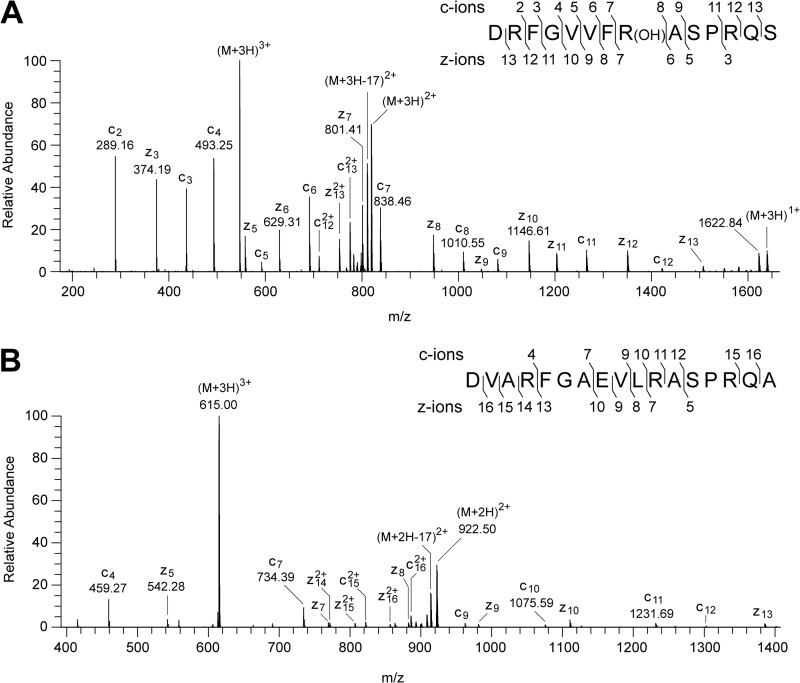
**Characterization of the hydroxylation of residue Arg-73 of the PSST subunit of human complex I and the unmodified residue in the *E. coli* NuoB subunit.** *A*, ETD fragmentation spectrum of a triply charged ion, *m*/*z* 546.62, generated by cleavage of the human PSST protein with Asp-N. The ions z_6_-z_7_ and c_7_-c_8_ show that the +16 Da modification is associated with residue Arg-73. *B*, spectrum of fragments produced by ETD from a triply charged ion (*m*/*z* 615.00) from a peptide corresponding to residues 77–93 of the *E. coli* NuoB subunit. The series of fragment ions identifies the peptide and excludes modification of residue Arg-87. In the *insets*, the fragment ions are mapped onto the amino acid sequence.

The digest of the bovine PSST subunit with protease Asp-N contained a hitherto unassigned peptide with *m*/*z* 1637.84. This value corresponds to a singly charged ion arising from residues 70–83 of the PSST subunit, with an additional mass of 16 Da. The spectrum of the fragments produced from the triply charged ion (Fig. 6) confirmed that the sequence of the peptide was DRFGVVFRASPRQS with an additional 16 mass units, corresponding to a hydroxyl group associated with residue Arg-77. Thus, this residue is probably hydroxyarginine. There was no evidence in any of the spectra for the unmodified form, and so it appears that this modification is quantitative. Residue Arg-77 is conserved in the human, *P. pastoris*, and *E. coli* enzymes (Fig. 7). An additional mass of 16 Da is associated with this residue in the human protein, and so Arg-73 also appears to be hydroxylated in the human PSST subunit (Fig. 8). The mass spectral data do not provide evidence about which atom in the side chain of bovine Arg-77 or human Arg-73 is hydroxylated. In the *E. coli* enzyme, the experimental and calculated molecular masses of the NuoB subunit are in agreement, and the equivalent arginine residue Arg-87 is unmodified (Fig. 8), and in the *P. pastoris* enzyme, the modification is not present either (26). It is not known whether the Nqo6 subunit of complex I from *P. denitrificans* is modified.

## DISCUSSION

### 

#### 

##### Characterization of Mammalian Complex I

Over the past 20 years, the chemical composition of bovine complex I has been studied and scrutinized in great detail, by characterization of cDNAs and especially by mass spectrometric analysis of its subunits (2, 10, 15, 23, 34, 35). Today, this enzyme is considered to be a complex of 44 proteins, seven of them being hydrophobic subunits encoded in the mitochondrial genome and the remainder being nuclear gene products. An important facet of the characterization of the chemical composition of complex I is the definition of the post-translational modifications of its subunits. None, either transient or permanent, has been detected in the seven mitochondrially encoded subunits with the exception of the transient phosphorylation of murine subunit ND5 (14), but many modifications of nuclear encoded subunits, both transient and permanent, have been reported (15–19, 22). Here, we have described the characterization of post-translational modifications by methylation and hydroxylation separately of two arginine residues, one in the 49-kDa subunit and the other in the PSST subunit of mammalian complex I. Both modifications are apparently quantitative.

##### Methylation of Mitochondrial Proteins

A small subset of proteins in mammalian mitochondria is known to contain lysine residues that are trimethylated, evidently completely and stably, on their ϵ-amino groups. They are citrate synthase (36), ADP-ATP translocase (37), and the c-subunit of ATP synthase (38). However, the biological significance of these modifications is not understood. In a survey of methylation of arginine residues in the mitochondria of *Trypanosoma brucei*, 167 proteins were found to be modified, but the orthologue of the 49-kDa subunit, which is encoded in the mitochondrial genome in trypanosomes and plants, where the equivalent arginine residue is conserved, was not one of them (39). In another human cell-wide survey of arginine methylation, the only mitochondrial protein detected with a methylarginine residue was kynurenine oxoglutarate transaminase 3, but the extent of modification was not determined (40).

Protein arginine methylation in mammals has roles in signal transduction, transcription, RNA processing, translation, DNA repair, protein translocation, endosomal trafficking, the nuclear pore complex, cytoskeleton dynamics, and probably other cellular processes also (40, 41). The methylation reaction, using *S*-adenosylmethionine as the methyl donor, occurs on the terminal nitrogen atoms of the guanidino groups of the arginine residues, and ω-N^G^-monomethylarginine, asymmetric ω-N^G^,N^G^-dimethylarginine, and symmetric ω-N^G^,N^G′^-dimethylarginine have all been found. Here, the 49-kDa subunit of bovine complex I has been shown unambiguously to be dimethylated; it contains symmetric ω-N^G^,N^G′^-dimethylarginine, at Arg-85, and the modification is conserved in the human, *P. pastoris*, and *P. denitrificans* orthologues. There was no evidence of the monomethylation of Arg-290, which has been reported to be monomethylated in the rat enzyme (42). In the majority of cases of methylation of arginine residues, the modified residue is followed by a glycine residue (39, 40), as it is in the 49-kDa subunit (Fig. 4).

Protein arginine methyltransferases (PRMTs) fall into three classes known as types I–III. All three types are associated with the production of ω-N^G^-monomethylarginine, and in addition, the type I enzymes produce asymmetric ω-N^G^,N^G^-dimethylarginine, but only the type II enzyme produces symmetric ω-N^G^,N^G′^-dimethylarginine (41). Hence, the modification of the 49-kDa subunit is likely to be catalyzed by a type II enzyme. The only known member of this class, PRMT5 (43), is conserved in all major animal groups and fungi and functions in both the cytoplasm and the nucleus, where it acts on many different protein substrates (44, 45). The cellular site of methylation of the 49-kDa subunit is not known. Its precursor form with the N-terminal import sequence could be methylated by PRMT5, or a relative, in the cytoplasm, although the 49-kDa subunit was not detected in a screen of methylation of arginine residues in total cellular proteins (40), or alternatively, it could be methylated in its mature form after import into the matrix of the organelle. No PRMT was detected by proteomic analysis of mouse mitochondria (46), but the possibility remains that the modification is catalyzed by an as yet uncharacterized mitochondrial PMRT. It is known that *S*-adenosylmethionine is transported into mitochondria by a specific carrier protein (47), and two mitochondrial proteins, C20orf7 (NDUFAF5) and MidA homolog NDUFAF7, may be methyltransferases. They are both required for the assembly of complex I (48, 49), and a yeast two-hybrid screen provided evidence of interaction between NDUFAF7 and the 49-kDa subunit in *Dictyostelium* (49).

##### Hydroxylation of Protein Arginine Residues

The hydroxylation of arginine residues is a rare post-translational modification found to date in only three proteins. A protein found in adhesive plaques of the mussel *Mytilus edulis* contains many 4-hydroxyarginine residues (50); the large subunit of carbon monoxide dehydrogenase from *Hydrogenophaga pseudoflava* has a 4-hydroxyarginine residue immediately preceding a catalytically essential cysteine residue (51); and the *E. coli* 50 S ribosomal protein L16 contains a single 3-hydroxyarginine residue produced by the oxygenase YcfD (52). On the basis of the experiments described here, it is not possible to identify precisely the site of hydroxylation of residue Arg-77 in the bovine PSST and the equivalent Arg-73 in the human protein. To do so might require isolating the hydroxyarginine residue from acid hydrolysates of the PSST subunit and then determining its structure by fast-atom bombardment mass spectrometry or by nuclear magnetic resonance experiments (50). It would probably be necessary to commit gram quantities of pure complex I to such an approach. Alternatively, if the modification were catalyzed by an identified oxygenase, it would be possible to determine its arginine hydroxylation specificity by hydroxylating and then characterizing model peptides (52).

##### Functional Significance of the Modifications of the 49-kDa and PSST Subunits

**FIGURE 9.**
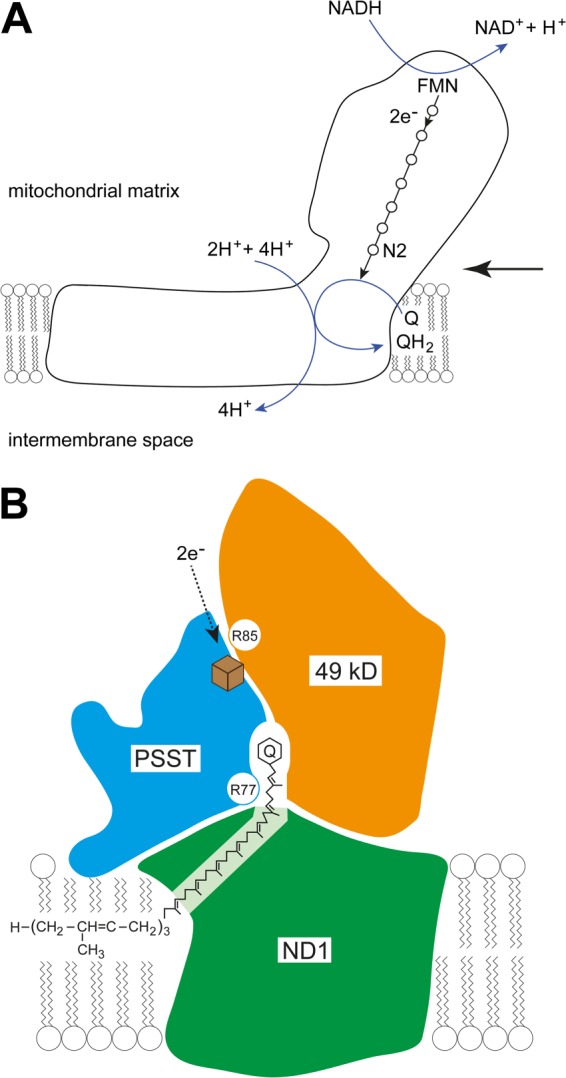
**Locations of the modified arginine residues in the vicinity of the quinone binding site of bovine complex I.** *A*, the outline of the structure of complex I illustrating the pathway of electron transfer from NADH to coenzyme Q via flavin mononucleotide and a chain of seven Fe-S clusters (*open circles*). The terminal Fe-S cluster N2 is labeled. As indicated, the transfer of two electrons one at a time via this pathway is coupled to the translocation of four protons into the mitochondrial intermembrane space from the mitochondrial matrix. The *large arrow* on the *right* indicates the direction of view of the quinone binding site in *B. B*, a detailed view of the quinone binding site involving the 49-kDa, PSST, and ND1 subunits (*orange*, *blue*, and *green*, respectively). The *green* ND1 subunit is divided into two unequal areas by the intervening quinone binding site, one small and approximately triangular, the other larger and approximately pentangular. The area shaded *lighter green* indicates that the two *dark green areas* are joined in front and behind the binding site. *B* is based upon the structures and positional relationships of the orthologous core proteins, Nqo4, Nqo6, and Nqo8, in the structure of complex I from *T. thermophilus* (5). The view is along the axis of the membrane arm of complex I away from its junction with the orthologous extrinsic arm (vertical pointing upward into the matrix of the mitochondrion). The approximate position of the phospholipid bilayer of the inner mitochondrial membrane is indicated. The quinone is shown with its head group and part of its side chain bound in a tunnel between the three subunits. The entrance to the tunnel is formed by the transmembrane α-helices 1 and 6 and amphipathic α-helix 1 of subunit Nqo8. The hydroxylated Arg-77 in the bovine PSST subunit is in a loop (not modeled in the structure of the bacterial complex, but linking α-helices 2 and 3). The dimethylated Arg-85 in the bovine 49-kDa subunit is in a loop between β-strand 3 and α-helix 1 close to Fe-S cluster N2 (*brown cube*), which is attached to the PSST subunit. The *arrow* indicates the direction of electron flow from the penultimate Fe-S cluster to the terminal cluster, N2.

Currently, there is no high resolution structural information about mammalian complex I, but the structure of complex I from the thermophilic bacterium *Thermus thermophilus* has been described (5), and it contains the structures of the orthologs of all of the core subunits of the mammalian complex, including those of Nqo4 and Nqo6, the orthologs of the 49-kDa and PSST subunit, respectively. The sequences of Nqo4 and the 49-kDa subunit and Nqo6 and the PSST subunit are 61 and 64% conserved, respectively, with 42 and 48% identical, respectively, (supplemental Fig. S1), and therefore, the structure of complex I from *T. thermophilus* provides a reasonable model for examining the general environments of the modified arginine residues in the 49-kDa and PSST subunits of the bovine and human enzymes. A schematic representation of the region where the modified residues are found is shown in Fig. 9. The dimethylated Arg-85 in the bovine 49-kDa subunit replaces a threonine residue in the bacterial enzyme in a loop between β-strand 3 and α-helix 1, close to (∼7 Å) Fe-S cluster N2, which is attached to the PSST subunit; the larger side chain of the methylated arginine could be even closer. The methylation of an arginine residue increases the hydrophobicity and solvent-accessible surface of the side chain, reduces its potential to form hydrogen bonds, and lowers its pI value slightly (53). Cluster N2 is the terminal Fe-S cluster in the chain of seven Fe-S clusters, and if for example the methylated arginine residue were close to one of the side chains of the cysteine residues that ligand the cluster, the dimethylated arginine residue might conceivably influence its redox potential. Another possibility is that the methylated residue influences the assembly of complex I, and the association of human pathogenic mutations in putative protein methylases that lead to dysfunction of complex I (48) makes this an attractive possibility. The hydroxylated Arg-77 in the bovine PSST subunit is in a loop (not resolved in the structure of the bacterial complex) linking α-helices 2 and 3 close to the tunnel, also involving the ND1 and 49-kDa subunits, in which oxidized coenzyme Q is thought to bind so as to accept electrons from cluster N2. In the absence of more detailed structural information, it is currently not possible to understand the role of the hydroxylated arginine residue. It is unlikely that the hydroxylation of the arginine residue arises in the tunnel, for example by reaction with reactive oxygen species, as such species are not thought to be generated from the quinone (54), although this conclusion is disputed (55). The restricted access to both modified residues would make enzymatic modification via the tunnel unlikely, and therefore, it is much more probable that the modifications occur in the cytoplasm at any one of the stages following synthesis, during or following import into the mitochondrion or during assembly into complex I. The identification of the modifying enzymes is likely to help in determining the cellular site of modification, and it may help also in understanding the roles of the modified arginine residues.

## Footnotes

2 The abbreviations used are:

CID: collision-induced dissociation
ETD: electron transfer dissociation
HCD: high energy collision dissociation
PRMT: protein arginine methyltransferase.
